# Molecular Markers of Pediatric Solid Tumors—Diagnosis, Optimizing Treatments, and Determining Susceptibility: Current State and Future Directions

**DOI:** 10.3390/cells11071238

**Published:** 2022-04-06

**Authors:** Joanna Trubicka, Wiesława Grajkowska, Bożenna Dembowska-Bagińska

**Affiliations:** 1 Department of Pathology, The Children’s Memorial Health Institute, Av. Dzieci Polskich 20, 04-730 Warsaw, Poland; w.grajkowska@ipczd.pl; 2 Department of Oncology, The Children’s Memorial Health Institute, Av. Dzieci Polskich 20, 04-730 Warsaw, Poland; b.dembowska@ipczd.pl

**Keywords:** pediatric solid tumors, molecular markers, prognostic and predictive marker, molecular target for therapy

## Abstract

Advances in molecular technologies, from genomics and transcriptomics to epigenetics, are providing unprecedented insight into the molecular landscape of pediatric tumors. Multi-omics approaches provide an opportunity to identify a wide spectrum of molecular alterations that account for the initiation of the neoplastic process in children, response to treatment and disease progression. The detection of molecular markers is crucial to assist clinicians in accurate tumor diagnosis, risk stratification, disease subtyping, prediction of treatment response, and surveillance, allowing also for personalized cancer management. This review summarizes the most recent developments in genomics research and their relevance to the field of pediatric oncology with the aim of generating an overview of the most important, from the clinical perspective, molecular markers for pediatric solid tumors. We present an overview of the molecular markers selected based on therapeutic protocols, guidelines from international committees and scientific societies, and published data.

## 1. Introduction

Recent decades have witnessed an intensive development of molecular research, which has contributed significantly to a more complete understanding of the molecular basis of childhood cancers. The published studies reveal a high heterogeneity of molecular alterations that account for the initiation of the neoplastic process, response to treatment and disease progression. These findings clearly indicate that the profile of molecular changes occurring in childhood malignancies differs significantly from the one observed in adult cancers. Thus, it is not possible to directly extrapolate the knowledge and experience with molecular markers from adults to the pediatric population. The differences observed pertain not only to the molecular basis, but also to the disease itself, its anatomical site and its histopathological features demanding the development of a different diagnostic and therapeutic approach for this group of patients.

Knowledge of specific childhood cancer genetic alterations present in tumor cells (somatic markers) as well as germline alterations is useful throughout the entire diagnostic and therapeutic process. There are genetic markers specific for histological types of cancer which are necessary for making a correct diagnosis (a group of diagnostic markers). Furthermore, there are molecular markers that correlate to the tumor’s grade, allowing us to predict the course of disease and prognosis (a group of prognostic markers) as well as to predict responses to a specific treatment (a group of predictive markers). Most promising are the molecular markers for targeted therapies. Extensive research on the biology of cancers, including their molecular profile, has influenced the current way of practicing medicine in the field of cancer diagnosis and treatment as well as the development of the so-called “personalized or precision medicine”. In recent years, there has been an attempt to change the paradigm of anticancer treatment, which assumes that the occurrence of specific molecular alterations may determine the efficacy of the administered treatments irrespective of the site and histological type of the tumor.

In addition to the changes occurring in the neoplasm itself, which are used as targets for therapy, alterations identified in the genetic material from patients’ peripheral blood (germline alterations) are also important. They allow us to determine whether the occurrence of a particular cancer is caused by the presence of a molecular defect. If the identified molecular alteration may have been inherited, assessing its presence in other family members enables us to identify individuals at a higher-than-average risk of developing cancer. These individuals should be screened systematically for early cancer detection. Germline alterations can also constitute prognostic and predictive markers.

Childhood cancers are rare, but they remain the second-leading cause of death in the pediatric population. Their incidence is 14–15 cases per 100,000 per year. They account for about 1–1.5% of cancers in the general population. Solid tumors account for approximately 60% of all childhood malignancies. Their broad spectrum includes [1,2,3]:

- Central nervous system (CNS) tumors (~20–23% *),
- Neuroblastoma (8–10% *),
- Wilms tumors, (7–8% *),
- Malignant bone tumors, (osteosarcoma and Ewing tumor) (~7% *),
- Soft tissue sarcomas (~7% *),
- Germ cell tumors (3–6% *),
- Hepatoblastoma, rarely hepatocarcinoma (0.5–2% *),
- Retinoblastoma (2.5–3% *),
- Other malignant epithelial neoplasms and malignant melanomas
- Other and unspecified carcinomas.

* of all malignant neoplasms of developmental age

Since the mid-1970s, the cure rates for most pediatric solid tumors have increased by as much as 50% [4]. At present, more than 80% of children with cancer are cured. These excellent cure rates are achieved with multidrug chemotherapy combined with surgery and/or radiotherapy in the case of solid tumors. However, there is not much to offer to children with refractory or relapsed disease after first- or second-line treatment. For these patients, innovative and effective medicines are needed.

In recent decades personalized treatments based on molecular markers have been developed for adults with cancer. Nevertheless, not enough progress has been made in the development and authorization of targeted therapies for childhood malignancies. Though molecular markers are routinely used in diagnosis, establishing risk groups in pediatric oncology, and novel medicinal products are being developed, with some exceptions, few breakthrough medicines have come to the market for children. Only few such medicinal products received marketing authorization for the treatment of pediatric malignancies. Among them are ABL-class inhibitors for Philadelphia positive acute lymphoblastic leukemia, anti-CD antibodies for B cell lymphomas, and anti-GD2 antibodies for children with high-risk neuroblastoma. Recently, Larotrectinib for children with *NTRK* fusion solid tumors and Crizotinib for children and young adults with relapsed or refractory systemic anaplastic large-cell lymphoma received marketing authorization. Phase 2/3 studies with Crizotinib in children and adolescents with recurrent, progressive, and unresectable inflammatory myofibroblastic tumors are forthcoming. 

Moreover, genetic data are lacking on the difficulty to treat refractory or relapsed solid tumors, limiting our knowledge of the molecular composition of such entities. To further improve cure rates in pediatric cancer it is essential to continue research and targeted medicine trials using tumor molecular profiling in children and adolescents. 

This article describes a spectrum of molecular markers of clinical relevance in pediatric solid tumors. The markers were selected based on therapeutic protocols, guidelines from international committees and scientific societies, and a review of the current literature.

## 2. Molecular Markers of Clinical Significance in Childhood Solid Tumors

### 2.1. Central Nervous System Neoplasms

Central nervous system (CNS) neoplasms are the most common solid tumors in children and a leading cause of childhood cancer-related deaths. Moreover, out of all survivors of childhood cancer, patients cured from CNS tumors present with the most severe treatment-related health conditions. At present, CNS neoplasms are the greatest challenge for pediatric oncology in its broad diagnostic and therapeutic aspects. 

Nevertheless, it is molecularly the best-understood group of childhood solid tumors. The results of multi-omics research led, amongst other things, to the definition of molecular subgroups in medulloblastoma, which have solid clinical implications (e.g., the WNT-activated medulloblastoma is associated with good prognosis and is the subject of de-escalation therapy trials, reducing late effects of treatment). There are also other CNS tumors with molecularly defined subgroups.

The clinical validity of molecular markers in diagnostic management has already been demonstrated in the WHO 2016 classification of central nervous system tumors, and their role was further emphasized in the guidelines prepared by an international consortium cIMPACT-NOW (The Consortium to Inform Molecular and Practical Approaches to CNS Tumor Taxonomy). In the latest WHO 2021 classification of tumors of the CNS, molecular markers are fundamental for making a proper diagnosis [5].

At the same time, there has been an increase in the number of molecularly targeted drugs in cancer. The selection of optimal therapy absolutely requires the assessment of specific molecular alterations (point mutations, amplifications, fusions, chromosomal rearrangements).

A set of clinically relevant molecular markers for pediatric central nervous system tumors is provided in Table 1 [5,6,7,8,9,10,11,12,13,14,15,16,17,18,19,20,21].

### 2.2. Neuroblastoma

Neuroblastoma (NBL) is the most frequent pediatric extracranial tumor originating from neural crest progenitor cells. It accounts for approximately 10% of all childhood malignancies and for up to 15% of deaths in children from cancer [22]. It is the most common cancer diagnosed in children under 12 months of age with a median age at diagnosis of 17 months [23]. Neuroblastoma can present along the sympathetic nervous system, with the most common abdominal location in the adrenal gland or sympathetic chain. It is a heterogenous disease which is reflected in its clinical course of spontaneous regression, differentiation or rapid progression despite intensive multimodal treatment. Patients with low- and intermediate-risk neuroblastoma have good prognosis, with cure rates over 85%, while the survival of children with high-risk disease is less than 50%.

The most significant prognostic factors in NBL are the child’s age at diagnosis, disease stage [4], tumor histology, DNA ploidy, *MYCN* amplification status and the presence of characteristic chromosomal aberrations (Table 2).

Recommendations are based on current therapeutic protocols and diagnostic guidelines from the European Neuroblastoma Group (SIOPEN group) and the INRG (International Neuroblastoma Risk Group Biology Committee) [24].

A set of molecular markers clinically relevant for neuroblastoma is provided in Table 2 [24,25,26,27,28].

### 2.3. Renal Tumors

Wilms tumor (nephroblastoma, WT) is the most common pediatric renal malignancy, accounting for over 90% of renal tumors. Other less frequently occurring malignancies of the kidney include:

- clear cell sarcoma of kidney (CCSK),
- renal cell carcinoma associated with MiTF/TFE translocations,
- malignant rhabdoid tumor of kidney (MRTK),
- congenital mesoblastic nephroma (CMN), and others.

The incidence of Wilms tumor is approximately 7 cases per 1 million children younger than 15 years of age, accounting for 5–7 percent of all childhood malignancies. WT can occur in both kidneys (bilateral disease), found in 5–8% of cases. The mean age at diagnosis is 44 months in unilateral cases and 31 months for bilateral cases of Wilms tumor. Wilms tumor is rare in patients older than age 15. A total of 1.5% of cases occur in related family members. Approximately 5% of WTs are associated with known constitutional predisposition syndromes.

Pathogenic changes in suppressors that regulate growth, differentiation and proliferation of embryonic kidney tissue play an essential role in the pathogenesis of this cancer. Alterations in *WT1*, *CTNNB1* or *AMER1* (*WTX*) genes are observed in about one-third of Wilms tumor cases [29,30]. Other important genes that regulate miRNA processing, such as *DROSHA*, *DGCR8, DICER1* and *XPO5,* are also involved [31,32,33,34]. The third important group consists of certain genes, the expression of which plays a significant role in the early stages of kidney development, such as *SIX1* i *SIX2*, *EP300* (*CREBBP*)*, MLLT1, BCOR* and *MYCN*. Alterations in the *TRIM28* gene are associated with the epithelial subtype of Wilms tumor [35]. In anaplastic Wilms tumors, the presence of *TP53* gene alterations is noted. An observed loss of heterozygosity within chromosome regions 1p and 16q as well as an increase in the amount of genetic material within chromosome 1q are associated with an unfavorable prognosis [36,37,38]. 

Recommendations are based on guidelines from two groups: Children Oncology Group (COG), continuing work of The National Wilms Tumor Study Group (NWTS) and The International Society of Paediatric Oncology-Renal Tumors Study Group (SIOP-RTSG), as well as the UMBRELLA therapeutic protocol and literature data [39,40,41,42].

A set of molecular markers clinically relevant for pediatric renal tumors is listed in Table 3 [35,36,37,43,44,45,46,47].

### 2.4. Malignant Bone Tumors, including Osteosarcoma and Ewing Sarcoma

Malignant bone tumors account for 6% of all childhood malignancies. The estimated incidence rate is 0.8 per million. The most common are osteosarcoma (56%), followed by Ewing sarcoma (34%) and chondrosarcoma (10%). The diagnosis of primary bone tumors relies on clinicopathological and radiological correlation. The recommended molecular assays include among others assessment of the presence of translocations characteristic of Ewing sarcoma and chondrosarcoma, determination of the status of pathogenic variants in the *H3F3A* gene that are relevant for the diagnostics of giant cell tumor of soft tissue, as well as assessment of the presence of *MDM2, PRIM1*, and *CDK4* amplifications to differentiate lower grade osteosarcoma. The standard was prepared based on the current literature data [48,49,50,51].

A set of molecular markers clinically relevant for malignant bone tumors of childhood is listed in Table 4 [4,50,51,52,53,54,55,56,57,58,59].

### 2.5. Soft-Tissue Sarcomas (STS)

Soft-tissue sarcomas are a heterogeneous group of malignant neoplasms that arise from embryonic mesenchymal and neuroectodermal tissue. They include neoplasms of muscle, connective and vascular tissue. Most cases occur in children aged 2–6 years and adolescents older than 12 years. The incidence rate, regardless of gender, ranges from 0.2–1.0/100,000 per year.

The most common STS in the pediatric group (70%) is rhabdomyosarcoma (RMS), with the age of onset usually before 10 years. The most common type of RMS is embryonal RMS. However, it is important to identify the alveolar RMS subtype for therapeutic management due to its worse prognosis. Recently, sclerosing and spindle cell rhabdomyosarcoma was separated as a stand-alone pathologic entity, in which two subtypes are molecularly defined: the infantile subset with *VGLL2, TEAD1* and *SRF* fusion as well as the subset with p.L122R *MYOD1* gene pathogenic variant. The presence of *MYOD1* alteration is associated with poor outcomes and response to therapy [60].

The remaining neoplasms belong to the non-rhabdomyosarcoma soft tissue sarcoma NRSTS group, which is more common in older children and young adults. Synovial sarcoma and MPNST (malignant peripheral nerve sheath tumor) are the most common neoplasms in this group. Other pathological entities are much less prevalent in children.

The recommendations were prepared based on the current literature data. A set of clinically relevant molecular markers for pediatric soft tissue sarcomas is provided in Table 5 [4,58,59,61].

### 2.6. Germ Cell Tumors

Germ cell tumors (GCTs) are derived from germ cells at different stages of their differentiation. They present a wide variety of site, histologic, and biological diversity. GCTs account for approximately 3–6% of all malignancies in children. The most common age of onset is between 1 and 6 years. Patients with GCT have good prognosis with cure rates over 85%. 

Currently, the recommended molecular assays for germ cell tumors include karyotyping of patients with symptoms of gonadal dysgenesis, gonadoblastoma and mediastinal tumors (for boys) and bilateral ovarian tumors (for girls), in order to detect/rule out the presence of genetic syndromes such as Turner, Swyer, Klinefelter, Fraser, Denys-Drash and others. The literature data also indicate the presence of somatic rearrangements involving chromosomes 1, 6, 11, 12, 16, 20, and 22, the clinical significance of which is currently unknown.

The standards were developed following the guidelines of the French TGM-95 protocol (1995), protocols elaborated by the international expert consortium MaGIC-Malignant Germ Cell International Collaborative and literature data [62]. 

### 2.7. Liver Tumors

The most common pediatric liver tumor is hepatoblastoma. The incidence of hepatoblastoma is 2–3 cases per 1 million children under 15 years of age; however, approximately 98% of all cases occur in children under 5 years of age. The mean age at diagnosis is 18 months [63]. Survival rates for children with hepatoblastoma exceed 80%. Molecular alterations reported in hepatoblastoma primarily involve genes, the protein products of which regulate the WNT and NF-κB pathway. Thus, the most common somatic alterations are those in genes: *CTNNB1* (80–90%), *APC* (2–3%), *AXIN1*, *AXIN2* and *PIK3CA* as well as *TERT* (2–6%) and *NFE2L2* (5–10%) [63,64,65,66,67,68,69]. The results of chromosomal rearrangement analysis indicate that this group of cancers has a higher rate of somatic rearrangements within chromosomes 1, 2, 8, and 20. However, the clinical significance of their occurrence is still unknown [64,65,70]. One of the objectives of the Pediatric Hepatic International Tumor Trial (PHITT (NCT03017326)), which has been running since 2017, is to determine the clinical significance of molecular findings including complete genomic, transcriptomic, and epigenomic profiling for hepatoblastoma patients [71].

Other hepatic tumors of childhood include: hepatocellular carcinoma (HCC) which is very rarely diagnosed in children, usually in older patients (10–14 years), but has been also found in children younger than 5 years of age. The prognosis is dismal, after conservative surgical treatment (30% of children achieving 3-year survival). Liver transplantation in children with HCC contributed to the improvement of overall survival (>70%). The most frequently reported somatic alterations in this cancer are alterations in the *TERT* and *TP53* genes (60% and 25–30%, respectively); however, the molecular background is still unknown [72,73];

- undifferentiated embryonal sarcoma of the liver (UES) which is a rare liver tumor with onset in children mostly aged between 6 and 10 years. The molecular background of this neoplasm is not fully understood; however, according to literature data, the characteristic features of this neoplasm comprise frequent and extensive chromosome rearrangements, also in the form of chromothripsis [74]. Additionally, alterations were observed within the 19q13.4 region, including a t(11;19) (q13;q13.4) translocation and overexpression of the C19MC region (miRNA cluster). The presence of *TP53* gene alterations was also observed [75,76,77,78].

The diagnostic standards for hepatoblastoma according to the International Childhood Liver Tumors Strategy Group (SIOPEL) were included in forming these guidelines. A set of clinically relevant molecular markers for hepatic tumors of childhood age is provided in Table 6 [64,70,78,79,80].

### 2.8. Retinoblastoma

Retinoblastoma is the most common primary malignant intraocular cancer in children and the second most common cancer of the eye in all age groups after choroidal melanoma. It accounts for 3% of all childhood tumors. The number of cases ranges from 1 in 14,000–1 in 18,000 live births [3,81]. The following forms of retinoblastoma are distinguished as:

- bilateral or multifocal (25–30% of cases, hereditary form),
- unilateral or unifocal (70–75% of cases, sporadic form),
- trilateral form, in which the presence of bilateral disease is accompanied by an embryonic intracranial tumor (pineoblastoma) localized in the midline (4%—only in children with the hereditary form of the disease).

Most cases are diagnosed between the ages of 1 and 3 years. The bilateral form of retinoblastoma is diagnosed earlier, before the age of 1. Rarely, the disease is diagnosed after the age of 5 years.

This cancer is associated with high (85–95%) penetrance *RB1* gene alterations [81]. There are recent reports of molecular alterations in retinoblastoma patients in genes other than *RB1*—Table 7 [82,83,84,85].

### 2.9. Melanoma

Melanoma is a malignant neoplasm of the skin, mucous membranes, or the choroid of the eye originating from melanocytes. The incidence is 1 case per 1 million children under 15 years of age. In the pediatric group, melanoma can present with one of three types:

- Spitzoid melanoma (SM), the most common form,
- Melanoma that arises from a congenital melanocytic nevus (CMN)
- Classic melanoma (“adult-type melanoma”), most similar in terms of causes and risk factors to melanoma diagnosed in adults.

Most commonly, melanoma is associated with molecular changes in genes that regulate the MAPK pathway. The recommendations were prepared following the current literature data. A set of molecular markers clinically relevant for childhood melanoma is provided in Table 8 [86,87,88,89,90,91].

### 2.10. Ovarian Cancers

The occurrence of ovarian cancer in girls may be associated with syndromes such as DICER1 or RTPS (rhabdoid tumor predisposition syndrome); therefore, both germ cell and somatic alterations are also observed in *DICER1* (sertoli-Leydig cell tumors) and *SMARCA4* (primary small cell carcinoma of the ovary, hypercalcemic type-SCCOHT) (Table 9) [92].

## 3. Targeted Treatments for Pediatric Solid Tumours

Treatments for pediatric malignancies have changed vastly over the last several decades and cure rates now reach over 80%. However, there are still children with uncurable malignancies and those who are cured experience treatment related chronic health conditions. The progress in the field of molecular biology, the ability to analyze tissue on genome-wide scales, to identify cancers with specific gene alterations with the intent to develop novel targeted treatments has created new opportunities to further improve survival of childhood cancer patients and their quality of life. To date the use of targeted and immune- therapies in children has been limited. Despite many obstacles of drug development in pediatric oncology some medicinal products have come to the market and are used in front-line treatment. There are ongoing pediatric phase I/II biomarker-driven trials in most difficult to treat solid tumors in children. Table 10 presents selected targeted treatments authorized or in development.

## 4. Germline Alterations

In recent years, we have been witnessing great progress in understanding the molecular profile of childhood cancers and applying this knowledge to clinical practice. This includes somatic as well as germline alterations. The published findings of different multi-omics studies further highlight the differences between childhood and adult cancers. The global number of somatic alterations, as expressed by the Tumor Mutational Burden (TMB), in childhood malignancies is much lower than in adults. With respect to germline alterations, the opposite is true. The occurrence of childhood cancers is more often determined by the presence of alterations responsible for genetic syndromes. More than 200 such syndromes have been identified, and this number is steadily growing. The fact that congenital cancers are also diagnosed underscores the significance of germline alterations in the pathogenesis of childhood cancers. It is estimated that approximately 7–8% of hematologic malignancies and solid tumors in children are determined by germline alterations [93]. Furthermore, these data appear to be underestimated since germline mosaicism, which is difficult to identify, or epigenetic changes, such as loss of imprinting of the 11p15 region or hypermethylation of the *CDKN2A* suppressor, are rarely assessed in the routine diagnosis of cancer. 

Assessment of the presence of germline alterations is also important in optimizing therapeutic management. If patients present with lesions that result from chromosomal instability or that occur in DNA repair genes, the omittance of radiotherapy or a reduced radiation dose are recommended. One such example is children with choroid plexus carcinoma and germline alterations in *TP53* gene. Patients with germline *RB1* alterations who underwent radiotherapy have twice the risk of developing secondary cancers compared with patients who did not receive radiotherapy [94]. The presence of germline alterations in the *NF1* gene in patients with low-grade gliomas is associated with a better prognosis; hence, the treatment undertaken may be less aggressive [95]. However, patients with germline alterations in genes belonging to the (MMR mismatch repair system) such as *MLH1*, *MSH2*, *MSH6*, and *PMS2* with brain cancers require more aggressive chemotherapy [96]. The presence of a germline alterations may also contribute to the earlier cancer onset. Rhabdoid tumors occur more frequently in patients under 4 years of age; however, the mean age of onset in patients with germline *SMARCB1* alterations is 6 months [97,98]. Therefore, it seems highly appropriate to introduce the assessment of germinal alteration status into the algorithm of diagnostic and therapeutic management of pediatric cancers.

The following tables (Table 11 and Table 12) present selected genetic syndromes associated with the occurrence of childhood cancers, as well as information about which genes should be assessed in selected cancers of this age group.

## 5. Material and Conditions for Its Preservation for Genetic Testing

The starting point for most oncogenetic tests used is a tumor tissue sample. Formalin fixation and paraffin embedding (FFPE) is the most common form of tissue preservation; however, for NGS-based protocols, tissue preservation by freezing is much more beneficial. In selected tumors (e.g., neuroblastoma), the impression smear of tumor tissue may serve as the material for genetic assay. To identify germline alterations, the patient’s peripheral blood, saliva and/or buccal swab samples are also collected in addition to the tumor tissue.

Due to the heterogeneous nature of tumors (particularly neuroblastoma and selected gliomas), it is recommended that at least two specimens be collected from the respective tumor tissue. In cases of tumor recurrence and probable changes in the molecular profile of the relapsed tumor tissue, resampling is indicated.

Each tumor tissue specimen for molecular assay should be evaluated for the percentage of tumor cells in the tested specimen. This assessment is routinely performed by a pathologist. If more than one biological sample is available, the most appropriate sample should be selected based on the type of molecular assay planned, the availability of the biological material and the need for it at subsequent stages of diagnostic process.

It is also very important to maintain sterility when collecting material for molecular assays. NGS methods can detect mosaic-type alterations at very low levels. In cases of contamination of the specimen with even a very small amount of material from another patient, false results may be obtained.

## 6. Future Directions

### 6.1. Methylation Profile

The rapid development of high-throughput next-generation sequencing methods has significantly contributed to the understanding of the molecular profile of the most common pediatric cancers. Today, we know that, on the one hand, these tumors exhibit a great variety of molecular alterations, but, on the other hand, their total number is small compared to the number of somatic alterations detected in tumors occurring in adults. This is reflected very frequently in the low TMB score, which translates into limited applicability of immunotherapy. Effective immunotherapy can also be limited by the suppressive tumor microenvironment with relatively few effector cells. Generally, this type of therapy in pediatric solid tumors still remains in the early stages of development and significant clinical benefit has yet to be demonstrated.

In contrast, epigenetic changes occupy a special place in the vast spectrum of molecular alterations that are identified in childhood cancers. It seems that this type of alteration is crucial for the initiation of carcinogenesis-related processes. A confirmation of this assumption is the occurrence of different genome DNA methylation patterns in different tumor subtypes. A unique epigenetic signature that represents both the tumor origin and the presence of acquired oncogenic alterations affecting chromatin state constitutes a very promising diagnostic tool for, among others, central nervous system tumors in children. The classification system developed by German National Cancer Institute (DKFZ) in Heidelberg, based on the methylome pattern [99] is slowly becoming a routine tool to accurately classify CNS tumors into distinct molecular subtypes. As a result, it may improve the accuracy of diagnosis and standardize pathomorphological assessment. This diagnostic approach is limited by the requirement for specialized equipment, software, and a large reference database. To address this problem, the DKFZ team developed a free online tool, Classifier [100], which allows for the processing of data obtained in a given laboratory and comparing them to a reference database containing the results of methylation profile analysis from over 2800 cases. An additional advantage of this solution is the ability to verify the histopathological diagnosis in morphologically ambiguous cases and, in the future, also the ability to identify new, very rare tumor subtypes, not only of the central nervous system.

### 6.2. Liquid Biopsy

There is a growing number of published studies demonstrating the important role of liquid biopsy in the diagnostic and therapeutic management of a number of cancers, including pediatric malignancies. It is a complementary or alternative method to surgical biopsy, as well as a non-invasive, promising tool for early cancer detection, that may also overcome problems of tumor accessibility and heterogeneity of tumor tissue. Various biological fluids, including peripheral blood, urine, cerebrospinal, synovial and ocular space fluids, can be used to obtain such circulating material as tumor cells (CTCs), tumor DNA (cfDNA), RNA (cfRNA), proteins and extracellular vesicles (EVs) for diagnostic assays. Thanks to the advances in technology, it is possible to obtain and analyze such biological material with increased effectiveness. There are data demonstrating the usefulness of liquid biopsy in variety of analysis including small- and large-scale mutation analysis, high throughput sequencing technologies, and analysis of structural or copy number alterations. From a clinical perspective, the results from liquid biopsy can provide reliable data as to the status of the disease and allow us to monitor treatment and to evaluate predictive, prognostic and resistance markers. In some cases, it may be helpful in early detection of recurrence. Thus, liquid biopsy, although still a relatively new method, appears to be a significant application for cancer diagnosis and treatment. The results of published research on neuroblastoma, sarcoma, Wilms tumor, hepatoblastoma and retinoblastoma appear to be very promising [101]. Nevertheless, the implementation of liquid biopsy into clinical practice is still to be completed. Its limitations are mainly due to the lack of standardized, validated methods for such analyses and the rarity and instability of obtained tumor biomolecules. However, researchers agree that liquid biopsy represents a potentially major new method that can be used to detect, monitor and treat cancers. Further studies are required to address the limitations of this technique. 

## 7. Conclusions

The introduction of technologies such as massively parallel DNA sequencing and RNA sequencing, as well as tools for the interpretation of the vast amounts of data obtained with these methods, including bioinformatic or crystallographic methods, creates an opportunity to elucidate the molecular mechanisms of childhood cancers and to develop targeted therapies. Artificial intelligence methods are also becoming increasingly employed to design therapeutic algorithms and identify prognostic and predictive markers [102]. The introduction of monitoring of circulating tumor DNA (ctDNA) using next-generation sequencing will enable future precise monitoring of treatment. The integration of a broad spectrum of data from “-omics” studies provides the basis for the development of cancer-specific classifiers used for precise diagnostics. More novel in vivo and in vitro models as well as 3D cultures are being developed and used to test drugs specifically dedicated to pediatric cancers. The aim of all of these efforts is to identify molecular markers and move them into the clinical setting for more precise diagnosis, risk stratification, and more effective and less toxic treatment in this therapeutically challenging group of patients.

## Figures and Tables

**Table 1:** Molecular markers—central nervous system tumors of childhood.

Tumor Type		Genes/Molecular Profiles Characteristically Altered	Diagnostic Marker	Prognostic, Predictive Marker, Target for Therapy
Gliomas, glioneuronal tumors, and neuronal tumors				
Pediatric-type diffuse low-grade gliomas	Diffuse astrocytoma, *MYB*- or *MYBL1*-altered	*MYB* *MYBL1* IDH-wild type (*IDH1, IDH2*) H3-wild type (*H3-3A, HIST1H3B, HIST1H3BC*)	+	Alterations involving *MYB* and *MYBL1* genes: favorable prognostic factor
	Angiocentric glioma	*MYB* (usually *MYB:QKI*)	+	Favorable prognostic factor
	Polymorphous low-grade neuroepithelial tumor of the young	*BRAF* FGFR family	+	Potential targets for tyrosine kinase inhibitors (depending on the alteration detected)
	Diffuse low-grade glioma, MAPK pathway-altered	*FGFR1* *BRAF*	+	Potential targets for tyrosine kinase inhibitors (depending on the alteration detected)
Pediatric-type diffuse high-grade gliomas	Diffuse midline glioma, H3 K27-altered	*H3-3A, HIST1H3B, HIST1H3BC*: p.K28M *TP53* *ACVR1* *PDGFRA* *EGFR* *EZHIP*	+	Pathogenic variants in genes encoding histone H3.3 - an unfavorable prognostic factor. Potential targets for targeted therapy (depending on the alteration detected)
	Diffuse hemispheric glioma, H3 G34-mutant	*H3-3A*: p.G35R/V *TP53* *ATRX* MGMT	+	MGMT - a favorable prognostic factor associated with increased sensitivity to temozolomide
	Diffuse pediatric-type high-grade glioma, H3-wildtype and IDH-wildtype (subgroups: pedRTK1, pedRTK2, pedMYCN)	IDH-wild type (*IDH1, IDH2*) H3-wild type (*H3-3A, HIST1H3B HIST1H3BC*) *PDGFRA* *MYCN* *EGFR* (methylome)	+	Potential therapeutic targets (depending on the alteration detected)
	Infant-type hemispheric glioma	*NTRK1/2/3* *ALK* *ROS1* *MET*	+	Potential target for tyrosine kinase inhibitors (depending on the alteration engraved)
Circumscribed astrocytic gliomas	Pilocytic astrocytoma	*KIAA1549-BRAF* *BRAF* *NF1* fusions involving *NTRK1* and *NTRK2* genes	+	Potential targets for tyrosine kinase inhibitors (depending on the alteration detected) *KIAA1549-BRAF*, *BRAF* alteration- a favorable prognostic factor
	High-grade astrocytoma with piloid features	*IDH1/IDH2* - wild type *EGFR* amplification wild type MAPK signaling pathway primarily: *BRAF* (mainly fusions), *NF1* *ATRX* *CDKN2A/B*, (methylome)	+	Potential targets for tyrosine kinase inhibitors (depending on the alteration detected)
	Pleomorphic xanthoastrocytoma	*BRAF* *CDKN2A/B*	+	Potential targets for tyrosine kinase inhibitors (depending on the alteration detected)
	Subependymal giant cell astrocytoma	*TSC1* *TSC2*	+	Potential targets for mTOR inhibitors
	Astroblastoma, *MN1*-altered	*MN1* (primarily a fusion with *BEND2*)	+	Favorable prognostic factor
Other	Ganglioglioma	MAPK signaling pathway: *BRAF* *RAS* *FGFR1/2* *RAF1* *NTRK2* *NF1*	+	Potential targets for tyrosine kinase inhibitors (depending on the alteration detected)
	Desmoplastic infantile ganglioglioma/desmoplastic infantile astrocytoma	MAPK signaling pathway: primarily *BRAF* alterations	+	Potential targets for tyrosine kinase inhibitors (depending on the alteration detected)
	Dysembryoplastic neuroepithelial tumor	*FGFR1*	+	Potential targets for tyrosine kinase inhibitors
	Rosette-forming glioneuronal tumor	*FGFR1* *PIK3CA* *NF1*	+	Potential therapeutic targets (depending on the alteration detected)
	Myxoid glioneuronal tumor	*PDFGRA*	+	-
	Diffuse leptomeningeal glioneuronal tumor	MAPK signaling pathway, primarily a fusion *KIAA1549-BRAF*, 1p structural rearrangements, (methylome)	+	Potential targets for tyrosine kinase inhibitors (depending on the alteration detected)
	Dysplastic cerebellar gangliocytoma (Lhermitte-Duclos disease)	*PTEN*	+	-
	Extraventricular neurocytoma	IDH-wildtype (*IDH1, IDH2*) *FGFR* (*FGFR1-TACC1* fusion)	+	-
Ependymal tumors				
Supratentorial ependymoma	Supratentorial ependymoma, *ZFTA* fusion-positive	**ZFTA* -RELA*	+	Fusion involving the *YAP1* gene - a favorable prognostic factor
	Supratentorial ependymoma, *YAP1* fusion-positive	*YAP1- MAMLD1*	+	Fusion involving the *ZFTA* gene - an unfavorable prognostic factor
Posterior fossa ependymoma	Posterior fossa ependymoma, group PFA	global reduction of H3 K27me3 (methylome)	+	Unfavorable prognostic factor
	Posterior fossa ependymoma, group PFB	H3 K27me3 (maintaining methylation levels) (methylome)	+	Favorable prognostic factors
Spinal ependymoma	Spinal ependymoma, *MYCN*-amplified	*NF2* *MYCN*	+	*MYCN* - an unfavorable prognostic factor
Choroid plexus tumors				
	Choroid plexus carcinoma	*TP53*	-	Unfavorable prognostic factor associated with reduced indications for radiotherapy
Embryonal tumors				
Medulloblastomas, molecularly defined	Medulloblastoma, WNT-activated	*CTNNB1* *APC*	+	Favorable prognostic factors
	Medulloblastoma, SHH-activated and *TP53*-wildtype	*TP53*- wild type *PTCH1* *SUFU* *SMO* *MYCN* *GLI2* (methylome)	+	Potential targets for SHH pathway inhibitors. Unfavorable prognostic factors (*MYCN*)
	Medulloblastoma, SHH-activated and *TP53*-mutant	*TP53* *PTCH1* *SUFU* *SMO* *MYCN* *GLI2* (methylome)	+	Potential targets for SHH pathway inhibitors. Unfavorable prognostic factors (*TP53, MYCN*)
	Medulloblastoma, non-WNT/non-SHH	*MYC* *MYCN* *PRDM6* (methylome)	+	
Other CNS embryonal tumors	Atypical teratoid/rhabdoid tumor	*SMARCB1* *SMARCA4* rearrangements of chromosome 22	+	*SMARCB1* - unfavorable prognostic factor, *SMARCA4* - a standard prognostic factor
	Embryonal tumor with multilayered rosettes	*C19MC- DICER1*	+	-
	CNS neuroblastoma, *FOXR2*-activated	*FOXR2*	+	-
	CNS tumor with *BCOR* internal tandem duplication	*BCOR*	+	unfavorable prognostic factor
Pineal tumors				
	Pineoblastoma	*RB1* *DICER1*	+	-
	Desmoplastic myxoid tumor of the pineal region, *SMARCB1*-mutant	*SMARCB1*	+	-

**Table 2:** Molecular markers—neuroblastoma.

Tumor Type	Genes/Molecular Profiles Characteristically Altered	Diagnostic Marker	Prognostic, Predictive Markers, Target for Therapy
Neuroblastoma	*MYCN* (amplification)	-	Unfavorable prognostic factor in patients older than 18 months at diagnosis. The presence of *MYCN* gene amplification is associated with a significantly higher risk of recurrence and death from progression.
	(NCA)—numerical changes in the number of chromosomes in the genetic material of cancer cells	-	Diploidy as observed in the genetic material of the tumor tissue is associated with an unfavorable course of the disease. In infants, hyperploidy is a favorable prognostic factor (it is associated with good response to chemotherapy).
	(SCA)—segmental chromosomal changes most commonly involving chromosome regions 1p, 1q, 2p, 3p, 4p, 11q and 17q	-	Most frequently observed in advanced stages of the disease in older children, unfavorable prognostic factors.
	*ALK:* - *SNP*(most frequent: p.F1174L, p.F1245C, p.R1275Q) - amplification - fusions	-	A potential target for ALK kinase inhibitors, unfavorable prognostic factor.

**Table 3:** Molecular markers—renal tumors of childhood.

Tumor Type	Genes/Molecular Profiles Characteristically Altered	Diagnostic Marker	Prognostic, Predictive Markers
Wilms tumor (nephroblastoma)	- *WT1* (11p13)	+	No clear data
	- LOH 1p/16q - *TP53*	-	Unfavorable prognostic factor
Congenital mesoblastic nephroma (CMN)	- t(12;15) *ETV6-NTRK3* - *EGRF-ITD*	+	-
Clear cell sarcoma of kidney (CCSK)	- *BCOR-* ITDs - t(10;17)(q22;p13) *YWHAE-NUTM2B* - t(12;22)(q13;q12) *EWSR1-AFT1* - *BCOR-CCNB3*	+	-
Renal carcinoma associated with MiTF/TFE translocations	- t(X;1)(p11.2;q21.2) *TFE3-PRCC* - t(X;17)(p11.2;q25) *TFE3-ASPL (ASPSCR1)* - t(X;1)(p11.2;p34) *TFE3-SFPQ (PSF)* and others	+	-
Malignant rhabdoid tumor of kidney (MRTK)	- *SMARCB1* - *SMARCA4*	+	Unfavorable prognostic factor
Metanephric tumors	- *BRAF* (p.V600E)	+	-

**Table 4:** Molecular markers—malignant bone tumors.

Tumor Type	Genes/Molecular Profiles Characteristically Altered	Diagnostic Marker
Osteosarcoma	*TP53* *RB1* 8q21-24 (amplification) *MDM2* (amplification) extensive and comprehensive chromosomal rearrangements	+
Ewing sarcoma	t(11;22)(q24;q12) *EWSR1-FLI1* t(21;22)(q12;q12) *EWSR1-ERG* t(2;22)(q33;q12) *EWSR1-CREB1* t(7;22)(p22;q12) *EWSR1-ETV1* t(17;22)(q12;q12) *EWSR1-E1AF* inv(22)(q12;q12) *EWSR1-ZSG* t(16;21)(p11;q22) *FUS-ERG* and others	+
Chondrosarcoma	*HEY1-NCOA2* t(1;5)(q42;q32) *RF2BP2-CDX1* *IDH1* *IDH2* *TP53*	+
Giant cell tumor of soft tissue	*H3F3A* *HRAS* *TP53*	+

**Table 5:** Molecular markers—soft tissue sarcomas.

Tumor Type	Genes/Molecular Profiles Characteristically Altered	Diagnostic Marker
Rhabdomyosarcoma Alveolar	t(2;13)(q35;q14) *PAX3-FOXO1* t(1;13)(p36;q14) *PAX7-FOXO1* t(2;2)(q35;p23) *PAX3-NCOA1* t(X;2)(q35;q13) *PAX3-AFX*	+
Rhabdomyosarcoma Embryonal	loss of heterozygosity 11p15, trisomy 2, 8, 11, 12, 13 and 20 pathogenic variants in RAS pathway genes (*NRAS, KRAS, HRAS, NF1, FGFR4*)	+
Rhabdomyosarcoma Sclerosing and spindle cell	*VGLL2, TEAD1, SRF fusion* *MYOD1* (p.L122R)	+
Synovial sarcoma	t(X;18)(p11,q11) *SS18-SSX1,* *SS18-SSX2,* *SS18-SSX4*	+
Malignant peripheral nerve sheath tumor	complex chromosomal aberrations, pathogenic alterations in *SUZ12* and *EED* genes, *NF1* inactivation	+
Alveolar soft-part sarcoma	t(X;17)(p11;q25) *ASPL(ASPSCR1)-TFE3*	+
Angiomatoid fibrous histiocytoma	t(12;16)(q13:p11) *FUS-ATF1* t(2;22)(q33;q12) *EWSR1-CREB1* t(12;22)(q13;q12) *EWSR1-ATF1*	+
*BCOR*—rearranged sarcoma	inv(X)(p11.4p11.22) *BCOR-CCNB3* t(X;4)(p11;q31) *BCOR-MAML3* t(X;22)(p11;q13) *ZC3H7B-BCOR*	+
*CIC*—rearranged sarcoma	t(4;19)(q35;q13) t(10; 19)(q26;q13) *CIC-DUX4* t(X;19)(q13;q13.3) *CIC-FOXO4*	+
Clear cell sarcoma	t(12;22)(q13;q12) *EWSR1-ATF1* t(2;22)(q33;q12) *EWSR1-CREB1*	+
Dermatofibrosarcoma protuberans	t(17;22)(q21;q13) *COL1A1-PDGFB*, ring chromosome r(17;22)	+
Desmoid-type fibromatosis	5q21 loss, trisomy 8, 20, pathogenic alterations in *CTNNB1* gene	+
Desmoplastic small round cell tumor	t(11;22)(p13;q12) *EWSR1-WT1*	+
Dedifferentiated Liposarcoma	ring and marker chromosome, 12q13-15: *MDM2, CDK4* region amplification	+
Epithelioid sarcoma	deletion 22q *SMARCB1* t(8;22)(q22;q11) t(10;22)	+
Epithelioid hemangioendothelioma	t(1;3)(p36;q25), *WWTR1-CAMTA1*, t(X;11)(p11;q22) *YAP1-TFE3*	+
Extraskeletal myxoid chondrosarcoma	t(9;22)(q22;q12) *EWSR1-NR4A3* t(9;17)(q22;q11) *TAF15 (TAF2N)-NR4A3* t(9;15)(q22;q21) *TCF12-NR4A3* t(3;9)(q11;q22) *TFG*-*NR4A3* fusion t(9;17)(q22;q11) *RBP56*-*NR4A3* fusion	+
Giant cell fibroblastoma	t(17;22)(q22;q13) *COL1A1-PDGFB*	+
Infantile fibrosarcoma	t(12;15)(p13;q25) *ETV6-NTRK3,* t(2;15)(p21;q25) *EML4*-*NTRK3*, *LMNA-NTRK1*, 1q deletion, trisomy 8, 11, 17, 20	+
Inflammatory myofibroblastic tumor	Translocations involving the 2p23 region; fusions involving the *ALK* gene (with multiple partner genes) t(3;6)(q12;q22) *TFG*-*ROS1*	+
Leiomyosarcoma	Complex aberrations, frequently with 1p deletion	+
Lipoblastoma	t(7;8) (q21q12) *COL1A2-PLAG1* del(8) (q12q24) *HAS2-PLAG1* t(8;14) (q12;q24) *PLAG1-RAD51L1* t(2;8) (q31;q12.1) *COL3A1-PLAG1*	+
Low-grade fibromyxoid sarcoma	t(7;16)(q33;p11) *FUS-CREB3L2* t(11;16)(p11;p11) *FUS-CREB3L1*	+
Mesenchymal chondrosarcoma	t(8;8)(q13;q21) *HEY1-NCOA2*	+
Myoepithelioma	t(6;22)(p21;q12) *EWSR1-POU5F1* t(1;22)(q23;q12) *EWSR1-PBX1* (19;22)(q13;q12) *EWSR1-ZNF444*	+
Myxoid round cell liposarcoma	t(12;16)(q13;p11) *FUS-DDIT3* t(12;22)(q13;q12) *EWSR1-DDIT3* (*CHOP*)	+
Myxoinflammatory fibroblastic sarcoma	t(1;10)(p22;q24) *TGFBR3/MGEA5*	+
Myxofibrosarcoma	ring chromosome	+
Solitary fibrous tumor	inv(12)(q13q13) *NAB2-STAT6*	+
Undifferentiated embryonal sarcoma of the liver	t(11;19)(q13,q13) *MALAT1-MHLB1*	+

**Table 6:** Molecular markers of liver tumors.

Tumor Type	Genes/Molecular Profiles Characteristically Altered	Diagnostic Marker	Prognostic, Predictive Markers
Hepatoblastoma	*CTNNB1*	+	-
	*APC*	+/-	-
	*NFE2L2*	-	unfavorable prognostic factor
Undifferentiated embryonal sarcoma of the liver (UES)	t(11;19)(q13;q13.4)	+	-
	the C19MC region amplification	+	-
Malignant rhabdoid tumor of the liver	*SMARCB1*	+	-

**Table 7:** Molecular markers—retinoblastoma.

Tumor Type	Genes/Molecular Profiles Characteristically Altered	Diagnostic Marker	Prognostic, Predictive Markers
Retinoblastoma	*RB1:* - SNPs: exons 1–27 of the gene including assessment of splicing sites and mosaic type changes - CNV - methylation of the promoter region	+	-
	*BCOR:* - SNPs - fusions	-	unfavorable prognostic factor
	*MYCN* (amplification)	-	unfavorable prognostic factor

**Table 8:** Molecular markers—melanoma.

Tumor Type	Genes/Molecular Profiles Characteristically Altered	Diagnostic Marker	Prognostic, Predictive Markers
Spitzoid melanoma (SM)	fusions involving *ROS1*, *NTRK3, NTRK3, ALK,* *BRAF, MAPK, MET, RET* genes	+	potential therapeutic targets
	segmental rearrangements within chromosomes	-	-
	homozygous deletion of the 9p21 region	+	unfavorable prognostic factor
	*TERT* (promoter changes-rare)	-	unfavorable prognostic factor
Melanoma arising from a congenital melanocytic nevus (CMN)	*NRAS* (most commonly p.Q61K/R)	+	potential therapeutic targets (depending on the alteration detected)
	*BRAF* (most commonly p.V600E)	-	
	*TERT* (promoter hypermethylation)	+	
	segmental rearrangements within chromosomes	-	
Classic melanoma (“adult-type melanoma”)	*BRAF* (most commonly p.V600E)	+	potential therapeutic targets (depending on the alteration detected)
	*TERT* (promoter changes)	+	
	segmental rearrangements within chromosomes	-	

**Table 9:** Molecular markers—ovarian cancers.

Tumor Type	Genes/Molecular Profiles Characteristically Altered	Diagnostic Marker	Prognostic, Predictive Markers
Sertoli–Leydig cell tumors	*DICER1*	+	-
Primary small cell carcinoma of the ovary, hypercalcemic type, SCCOHT	*SMARCA4*	+	-

**Table 10:** Selected targeted treatments for pediatric solid tumours—authorized or under development.

Specific Gene Mutation/Alteration	Targeted Treatment	Development Phase	Clinical Trial Identifier	Target Population
*ALK* alterations: - fusions - point mutations	AKL- inhibitors:	Phase I/II	NCT00939770	Anaplastic lymphoma kinase (ALK) positive tumors, relapsed or refractory solid tumors or anaplastic large cell lymphoma,
	Crizotinib	Phase II/III	NCT03874273	inflamatory myofibroblastic tumor
		Phase III	NCT03126916	neuroblastoma
		Phase II	NCT02034981	patients harboring an alteration on *ALK*, *MET* or *ROS1*
	Ensartinib	Phase II	NCT03213652	Relapsed or refractory advanced solid tumors, Non-Hodgkin lymphoma, or histiocytic disorders with *ALK* or *ROS1* alterations
Anti-CD 20 antibody	Rituximab	Authorized	-	Mature B cell Lymphoma
Anti-GD 2 antibody	Dinutuximab	Authorized	-	Neuroblastoma
Anti-CD-30 antibody	Brentuximab Vedotin	Phase III	NCT02166463 NCT01979536	Hodgkin Lymphoma ALCL
*BRAF* alterations: - point mutations (including p.V600E) - Fusions (*KIAA1549:BRAF*)	Dabrafenib	Phase I/II	NCT01677741	Advanced *BRAF* V600 mutation-positive solid tumors
	Dabrafenib + Trametinib	Phase II	NCT02684058	*BRAF* V600 mutation positive low grade glioma or relapsed or refractory high grade glioma
	Vemurafenib	Phase II	NCT03220035	Relapsed or refractory advanced solid tumors, Non-Hodgkin Lymphoma, or histiocytic disorders with *BRAF* V600 mutations, Langerhans cell histiocytosis (LCH), and other histiocytic disorders.
	Cobimetinib	Phase II	NCT04079179	Refractory langerhans cell histiocytosis (LCH), and other histiocytic disorders.
	Trametinib	Phase II	NCT03363217	Pediatric neuro-oncology patients with refractory tumor and activation of the MAPK/ERK pathway
	Selumetinib	Phase III	NCT04576117	Recurrent or progressive low-grade glioma
*CDKN2A/B* deletion	Palbociclib	Phase II	NCT03526250	Rb positive advanced solid tumors, Non-Hodgkin Lymphoma, or histiocytic disorders with activating alterations in cell cycle genes
	Ribociclib with Everolimus	Phase I	NCT03387020	Recurrent or refractory malignant brain tumors
*EZH2* alterations	Tazemetostat	Authorized	-	Epithelioid sarcoma ≥16 years
*FGRF* alterations	Erdafitinib	Phase II	NCT03210714	Patients with relapsed or refractory advanced solid tumors, Non-Hodgkin lymphoma, or histiocytic disorders with *FGFR* alterations
*H3-3A, HIST1H3B,* *HIST1H3BC* point mutation	Panobinostat	Phase I	NCT02717455	DIPG (H3K27M)
	Vorinostat	Phase II	NCT02035137	Neuroblastoma
	GD2 CART-cell	Phase I	NCT03635632	Relapsed or refractory neuroblastoma and other GD2 positive cancers
LSD1	Seclidemstat	Phase I	NCT03600649	Ewing or Ewing-related sarcomas
*MEK* alterations	Cobimetinib	Phase I/II	NCT02639546	Gliomas, sarcomas, neuroblastoma, melanoma, MPNST, rhabdoid tumors, including atypical teratoid/rhabdoid tumor (AT/RT), *NF1*-associated tumors or RASopathy-associated tumors
	Selumetinib	Phase III	NCT04576117	Recurrent or progressive low-grade glioma
		Authorized	-	Plexiform neurofibroma
*MET:* - amplifications - fusions	Volitinib	Phase I	NCT03598244	Recurrent or refractory primary CNS tumors
mTOR pathway genes alterations, including *TSC1*, *TSC2*	Everolimus	Authorized	-	Subependymal giant cell astrocytoma (SEGA)
	Temsirolimus	Phase III	NCT02567435	Rhabdomyosarcoma
*NTRK* gene fusions	Vitrakvi/Larotrectinib Entrectinib	Authorized	-	Treatment of adult and paediatric patients with solid tumours that display a neurotrophic tyrosine receptor kinase (*NTRK*) gene fusion
*PARP* alterations	Olaparib	Phase I	NCT04236414	Pediatric solid tumours
PD-1/PD-L1	Pembrolizumab	Authorized	-	R/R classic Hodgkin Lymphoma, melanoma ≥12 years
	Ipilimumab	Authorized	-	
	Pembrolizumab	Phase I	NCT02359565	Recurrent, progressive, or refractory high-grade gliomas, diffuse intrinsic pontine gliomas, hypermutated brain tumors, ependymoma or medulloblastoma
	Nivolumab	Phase II	NCT03173950	Medulloblastoma, ependymoma, choroid plexus tumors, atypical/malignant meningioma
*RET* alterations	Selpercatinib	Authorized	-	Treatment of adults and adolescents 12 years and older with advanced *RET*-mutant medullary thyroid cancer (MTC)
*ROS1* fusions	Repotrectinib	Phase I/II	NCT04094610	Pediatric and young adult subjects harboring *ALK, ROS1*, or *NTRK1/2/3*
	Entrectinib	Phase I/II	NCT02650401	Locally advanced or metastatic solid or primary CNS tumors
	Ensartinib	Phase II	NCT03213652	Relapsed or refractory advanced solid tumors, non-hodgkin lymphoma, or histiocytic disorders with *ALK* or *ROS1* alterations
*SMARCB1* - point mutations - CNVs	Tazemetostat	Phase I	NCT02601937	Rhabdoid tumors, INI1-negative tumors
*SMO* alterations	Vismodegib	Phase II	NCT01878617	Medulloblastoma SHH subtype
			NCT01601184	
	Sonidegib	Phase I/II	NCT01125800	Medulloblastoma, advanced pediatric solid potentially dependent on the Hedgehog-signaling pathway
*VEGFR, PDGFR* alterations	Pazopanib	Phase II	NCT01956669	Pediatric solid tumors
	Regorafenib	Phase II	NCT02048371	Selected sarcoma subtypes: (Ewing sarcoma, rhabdomyosarcoma, osteosarcoma)
*VEGFR1, VEGFR3, FGFR3, FGFR4, PDGFRA* alterations	Lenvatinib	Phase I/II	NCT02432274	Refractory or relapsed solid malignancies

**Table 11:** Selected genetic syndromes associated with childhood cancers.

Syndrome	Cancers	Gene/Chromosome Region
Li-Fraumeni	sarcomas, leukemias, brain cancers, hepatoblastoma	*TP53*
Xeroderma pigmentosum	melanoma	*XPA, XPC, DDB2, ERCC2*
Neurofibromatosis type 1	lymphomas, brain cancers, sarcomas, optic nerve gliomas, meningiomas, Wilms tumor, rhabdomyosarcoma	*NF1, SPRED1*
Ataxia-telangiectasia	CNS, GI tumors, leukemias	*ATM*
Bloom syndrome	acute leukemia, GI cancers	*BLM*
Fanconi anemia	acute leukemia, liver tumors	*FANCA, FANCB, FANCC, PALB2* and others
Nijmegen syndrome	leukemias, lymphomas, medulloblastoma, glioma, rhabdomyosarcoma	*NBN*
Beckwith-Wiedemann syndrome	nephroblastoma, hepatoblastoma, rhabdomyosarcoma, gonadoblastoma	*CDKN1C*/11p15
Chromosomal syndromes (Down syndrome, Klinefelter syndrome)	leukemias, CNS tumors	trisomy 21, 47XXY
Familial retinoblastoma	retinoblastoma	*RB1*
Familial Wilms tumor	nephroblastoma	*WT1*, *WT2* and others
Familial polyposis coli	hepatoblastoma	*APC, MUTYH*
Cardiofaciocutaneous syndrome (CFC)	acute lymphoblastic leukemia, rhabdomyosarcoma, hepatoblastoma, lymphomas	*BRAF, MAP2K1, MAP2K2, KRAS,*
Noonan syndrome	neuroblastoma, acute lymphatic leukemia, glioma, rhabdosarcoma	*PTPN11, RAF1, BRAF, SOS1, NRAS, CBL*
Costello syndrome	rhabdosarcoma, neuroblastoma, fibrosarcoma	*HRAS*
Sotos syndrome	Wilms tumor, neuroblastoma, hepatoblastoma	*NSD1*
Von Hippel–Lindau syndrome	renal tumors, CNS tumors-especially of the cerebellum, tumors of the adrenal glands, and tumors of the retina.	*VHL*
Gorlin syndrome	medulloblastoma	*PTCH1*
Rubinstein–Taybi syndrome	medulloblastoma, meningiomas, acute lymphatic leukemia, pheochromocytoma, rhabdomyosarcoma	*CREBBP*
Turcot syndrome	medulloblastoma, gliomas	*APC*
DICER syndrome	pleuropulmonary blastoma, nephroblastoma, renal and brain sarcomas, thyroid adenomas and carcinomas, gonadal tumors	*DICER1*
Multiple endocrine neoplasia type 1 and 2	adenomas/carcinomas of the endocrine system	*MEN1* and *RET*
Tuberous sclerosis	brain and kidney tumors	*TSC1* i *TSC2*
Trisomy 18	hepatoblastoma	trisomy 18
Simpson–Golabi–Behmel syndrome type 1	hepatoblastoma	*GPC3*
Glycogen storage disorder type 1a, III, IV, VI	hepatoblastoma	*G6PC, AGL, GBE1, PYGL*
Tyrosinemia type 1	hepatocellular carcinoma	*FAH*

**Table 12:** Germline alterations in selected solid tumors in children.

Tumor Type	Gene (MIM Number)
AT/RT	*SMARCB1* (MIM 601607) *SMARCA4* (MIM 603254)
Choroid plexus carcinoma	*TP53* (MIM 191170)
Congenital melanocytic nevi	*MC1R* (MIM 155555)
Familial melanomas	*CDKN2A* (*MIM* 600160)*,* *CDK4* (MIM 123829)
Glioma of the optic pathway	*NF1* (MIM 613675)
Hemangioblastoma	*VHL* (MIM 608537)
Malignant nerve sheath tumor	*NF1* (MIM 613675), *TP53* (MIM 191170)
Medulloblastoma	*APC* (MIM 611731) *BRCA2* (MIM 600185) *MLH1* (MIM 120436) *MSH2* (MIM 609309) *MSH6* (MIM 600678) *PMS2* (MIM 600259) *PALB2* (MIM 610355) *PTCH1* (MIM 601309) *SUFU* (MIM 607035) *SMOH* (MIM 601500) *TP53* (MIM 191170) *CREBBP* (MIM 600140) *GLI3* (MIM 175700)
Meningioma	*NF2* (MIM 607379) *PTCH1* (MIM 601309) *PTEN* (MIM 601728) *SMARCB1* (MIM 601607 *SMARCE1* (MIM 603111) *SUFU* (MIM 607035) *WRN* (MIM 604611) *MEN1*(MIM 613733)
Pineoblastoma	*DICER1* (MIM 606241) *RB1* (MIM 614041)
Schwannoma	*NF2* (MIM 607379) *PRKAR1A* (MIM 188830)
Schwannomatosis	*LZTR1* (MIM 600574) *SMARCB1* (MIM 601607)
Spinal cord ependymoma	*NF2* (MIM 607379)
Subependymal giant cell astrocytoma	*TSC1/TSC2* (MIM 605284/191092)
Neuroblastoma	*PHOX2B* (MIM 603851) *ALK* (MIM 105590)
Hepatoblastoma	*APC* (MIM 611731)*,* uniparental disomy at 11p15.5
Retinoblastoma	*RB1* (MIM 614041)

## Data Availability

No new data were created or analyzed in this study. Data sharing is not applicable to this article.
